# A LATENT CLASS ANALYSIS OF STRESSORS AND RESOURCES AMONG SPOUSAL CAREGIVERS TO OLDER ADULTS IN THE UNITED STATES

**DOI:** 10.1093/geroni/igac059.3106

**Published:** 2022-12-20

**Authors:** Ruotong Liu, Iris Chi, Shinyi Wu

**Affiliations:** Oregon Health & Science University, Marina del Rey, California, United States; University of Southern California, Los Angeles, California, United States; University of Southern California, Los Angeles, California, United States

## Abstract

Spousal caregivers to older adults experience differing intensities of co-occurring caregiving stressors and resources. Analyzing their heterogeneous profiles can improve recognition of spousal caregivers needing support and caregiving impact on their health. This study aims to identify latent classes among spousal caregivers to older adults and to explore the associated background characteristics and health outcomes. Using pooled data from Round 5 and Round 7 of National Study of Caregiving and the linked National Health and Aging Trends Study, eight indicators for stressors and three indicators for resources are included for latent class analysis to identify distinguishable spousal caregiver subgroups. ANOVA test or Chi-square test are used to understand the different characteristics across spousal caregiver classes. Multivariate regression is conducted to examine associations between class membership and health outcomes. Three latent classes are identified among the 793 included spousal caregivers: low-stress low-support class (39%), medium-stress high-support class (36%), and high-stress medium-support class (25%). Compared to the other two classes, high-stress medium-support class includes more female than male, medium-stress high-support class has greater portions of non-Hispanic Black individuals, and caregivers in low-stress low-support class have fewer comorbidities. In addition, compared to low-stress low-support class, spousal caregivers in high-stress medium-support class reported lower (0.42 unit) level of self-rated health and higher (1.87 unit) level of depressive symptoms. These results confirmed the heterogeneity among spousal caregivers, and highlighted the elevated needs to address the physical and mental health needs among spousal caregivers whose levels of resources could not offset the caregiving stress they experience.

